# A budget impact analysis of the introduction of erythropoiesis stimulating agent subsequent entry biologics for the treatment of anemia of chronic kidney disease in Canada

**DOI:** 10.1186/s40697-014-0028-3

**Published:** 2014-11-11

**Authors:** Nicole W Tsao, Clifford Lo, Marianna Leung, Judith G Marin, Dan Martinusen

**Affiliations:** Collaborations for Outcomes Research and Evaluation, Faculty of Pharmaceutical Sciences, University of British Columbia, 4102-2405 Wesbrook Mall, Vancouver, BC V6T 1Z3 Canada; British Columbia Provincial Renal Agency, 700-1380 Burrard St, Vancouver, BC V6Z 2H3 Canada; St. Paul’s Hospital, Providence Healthcare, Vancouver, BC V6Z 1Y6 Canada

**Keywords:** Erythropoiesis stimulating agents, Epoetin alfa, Darbepoetin alfa, Subsequent entry biologics, Biosimilars, Budget impact analysis, Economic analysis, Health care costs

## Abstract

**Background:**

In Europe, epoetin subsequent entry biologics (SEBs) have been in use since 2007. Canadian patents of erythropoietin stimulating agents are expiring in 2014, therefore it is predicted that epoetin SEBs will penetrate the Canadian market in the near future.

**Objective:**

To estimate the economic impact and costs offsets associated with the uptake and use of one or more epoetin SEBs in Canada for the treatment of anemia in chronic kidney disease.

**Design:**

A Canada-wide epidemiological-based budget impact analysis was conducted to estimate cost outcomes under two scenarios: with and without the availability of epoetin SEB.

**Setting:**

The analysis was conducted from the perspective of the Canadian healthcare payer, over a 5-year time horizon from 2015 to 2019.

**Patients:**

Patients included in the model were those with chronic kidney disease stages 3 to 5, who have renal anemia and require treatment with erythropoietin stimulating agents.

**Methods:**

Only direct medical costs pertaining to drug acquisition of currently available erythropoietin stimulating agents in Canada were incorporated in the model. Cost of epoetin SEBs, market shares, uptake rates, and other model inputs were estimated from published sources or databases. No discounting of future costs was applied.

**Results:**

Based on our analysis, under market phenomena similar to those seen in the Europe and without considering potential switching from originator epoetin to epoetin SEB, we could expect that Canadian adoption of epoetin SEBs could result in $35 million (2013 CAD, year 1) to $50 million (year 5) cost savings annually, with cumulative savings of $221 million after 5 years. In one-way sensitivity analyses, model variables with substantial impact on cost savings were the prevalence of chronic kidney disease and epoetin SEB uptake rates.

**Limitations:**

We did not take into account costs associated with overhead, administration, or adverse events.

**Conclusion:**

The advent of SEBs represents an opportunity to relieve financial pressure in our healthcare system. Under the assumption that the efficacy and safety of epoetin SEBs are non-inferior to originator products, we have shown that the adoption of epoetin SEBs can lead to cost savings for the Canadian payer.

## Abrégé

**Contexte:**

L’époétine biosimilaire est utilisée en sol européen depuis 2007. Puisque les brevets d’utilisation des agents stimulant l’érythropoïèse (ASE) viennent à échéance en 2014, il est à prévoir que l’époétine biosimilaire, qui est un produit biologique ultérieur, fera son entrée sur le marché canadien dans un avenir rapproché.

**Objectifs:**

Estimer l’impact économique et les coûts de compensation qui découlent de l’utilisation d’une ou de plusieurs formes d’époétines biosimilaires, dans le traitement de l’anémie associée à la maladie rénale chronique, au Canada.

**Type d’étude:**

Une analyse d’impact budgétaire pancanadienne basée sur des données épidémiologiques a été réalisée afin d’estimer les coûts reliés à deux scénarios éventuels: le premier avec époétine biosimilaire sur le marché, et le deuxième sans cette disponibilité.

**Contexte:**

L’analyse a été menée à partir de la perspective du contribuable au système de soins de santé canadien, sur un horizon de cinq ans, soit de 2015 à 2019.

**Participants:**

Le modèle ne tient compte que des patients atteints d’une maladie rénale chronique de stades 3 à 5, souffrant d’anémie reliée à la maladie rénale, et nécessitant un traitement avec des agents stimulant l’érythropoïèse.

**Méthodes:**

Les seuls coûts pris en compte par le modèle sont les coûts médicaux directs découlant de l’achat des agents stimulant l’érythropoïèse disponibles au Canada. Les coûts reliés à l’époétine biosimilaire, les parts de marché, les coûts de compensation, et les autres entrées du modèle ont été estimés à partir de sources publiées ou de bases de données. Aucune actualisation des coûts futurs n’a été estimée.

**Résultats:**

Selon notre analyse, qui ne tient pas compte de la possibilité d’utiliser l’époétine biologique de référence avant de passer à l’époétine biosimilaire, et sous des conditions de marché similaires à celles observées en Europe, l’adoption à l’échelle canadienne de l’époétine biosimilaire pourrait résulter en une économie de 35 millions $ (2013 $ CAN, année 1) à 50 millions $ (année 5) par année, pour une économie cumulative potentielle de 221 millions $ après cinq ans. L’analyse de sensibilités unidirectionnelles montre que la prévalence de la maladie rénale chronique et les coûts de compensation reliés à l’époétine biosimilaire sont les variables du modèle ayant le plus d’impact économique.

**Limites de l’étude:**

Nous n’avons pas tenu compte des frais généraux, d’administration, ou découlant d’imprévus.

**Conclusion:**

Les produits biologiques ultérieurs peuvent aider à alléger les difficultés financières de notre système de santé actuel. En supposant que l’époétine biosimilaire présente une efficacité et une innocuité qui ne sont pas inférieures à celles des époétines biologiques de référence, nous montrons que l’utilisation de l’époétine biosimilaire peut mener à des économies pour le contribuable canadien.

**What was known before:** Canadian patents for epoetin alfa and darbepoetin alfa are expiring, as such there is an opportunity to reduce expenditure on erythropoiesis-stimulating agents (ESAs) by adopting subsequent entry biologics (SEBs). In Europe, epoetin SEBs have been introduced at lower prices than innovator epoetins and have been in use since 2007.

**What this paper adds:** To date, published studies have only examined the budget impact of epoetin SEBs for the management of chemotherapy-induced anemia in European countries. This analysis takes the market experience of epoetin SEBs in Europe and models the economic impact of the introduction of these SEBs into the Canadian landscape. Our results serve to inform Canadian stakeholders including our policy makers, budget holders, researchers, and clinicians.

## Background

Over the last two and a half decades, the ESAs epoetin alfa (Eprex®) and darbepoetin alfa (Aranesp®) have become the standard of care for treating patients with anemia of chronic kidney disease (CKD) in Canada. With Canadian patents expiring in May 2014 and August 2014, respectively [1], there is an opportunity to reduce expenditure on ESAs by using “copies” of these innovator biologics called SEBs. In Europe, epoetin SEBs have been in use since 2007 [2]. Therefore, we predict companies will submit at least two epoetin SEBs to Health Canada for evaluation before the end of 2015. With no Canadian economic analyses published on this subject to date, we conducted a budget impact analysis (BIA) to estimate the financial impact of these agents in treating anemia of CKD in the Canadian setting.

A BIA is an essential part of economic evaluations for healthcare interventions. Reimbursement authorities increasingly utilize these analyses before making a listing or reimbursement decision on a new healthcare intervention. The impact on a particular budget, that is, the difference in healthcare expenditures before and after reimbursement or listing of the new intervention, is a commonly used measure of forecasting the fiscal impact. A BIA also can be used for budget planning and resource management within an organization. BIA addresses the expected changes in expenditures from the perspective of a payer, for a specified period of interest after adoption of the new intervention. The aim of a BIA is to provide a valid computing framework (a “model”) that allows users to apply input values pertinent to their setting to compute the possible financial and organizational consequences of coverage decisions for a new healthcare intervention [3].

This paper discusses the estimated economic impact and costs offsets associated with the uptake and use of one or more epoetin SEBs in Canada for the treatment of anemia of CKD. We also hope to inform clinicians, budget holders, hospital administrators and Pharmacy and Therapeutics Committee members across Canada as to the economic impact of the provision of epoetin SEBs in their respective institutions.

## Methods

### Model overview

We developed a budget impact model that estimated the economic consequences and costs offsets associated with the introduction of epoetin SEBs in Canada. The model was constructed in Microsoft Excel. The development of this model followed the Principles of Good Practice for Budget Impact Analysis II: Report of the International Society of Pharmacoeconomics and Outcomes Research (ISPOR) Task Force on Good Research Practices [3], wherever possible.

### Model description

The model was designed as a Canada-wide epidemiological-based analysis, and estimated economic outcomes under two scenarios:

1. ‘Reference scenario’: In the absence of epoetin SEBs for anemia of CKD treatment in Canada.
2. ‘SEB scenario’: Presence of one or more epoetin SEBs for anemia of CKD treatment in Canada.

Under the ‘Reference scenario’ arm, the eligible patient population for treatment with currently available ESAs and their associated resource utilization and economic impact were calculated. Under the ‘SEB scenario’ arm, we assumed that only a subset of the eligible population would receive treatment with epoetin SEBs, according to plausible estimates of market uptake.

### Time horizon

The budget impact model estimated the economic impact of adopting epoetin SEBs for anemia treatment over a five-year time horizon, with cycle lengths of one year. The model default base year is 2014 and the analysis estimated the budget impact in years 2015, 2016, 2017, 2018, and 2019.

### Perspective

The budget impact model was built from the perspective of the Canadian healthcare payer or budget holder. The base case assumption is that all patients in the model will be covered under the payer’s budget. Though in Canada, we do not have a pan-Canadian payer, this perspective can be adapted to regional payers or budget holders of local institutions.

### Study population

The analysis was performed for patients with CKD stages III to V, who have renal anemia and require treatment with ESAs. All populations who are candidates for ESAs were assumed to also be candidates for epoetin SEBs under the ‘SEB scenario’. The study population was derived from the projected Canadian population based on Statistics Canada [4] projections over years 2015 to 2019, and estimated using the disease incidence and prevalence of CKD stages III/IV not requiring dialysis, and CKD stage V patients requiring dialysis. Based on the literature, the prevalence of stage III/IV CKD in Canada is 3.04% and stage V CKD is 0.12%; the incidence of CKD was estimated to be 407.6/100,000 annually for stages III/IV and 19.2/100,000 annually for stage V [5-7].

Mortality rates of individuals in CKD stage III/IV are not well characterised in literature and databases. We estimated the mortality rate to be approximately 2% annually for this cohort based on a retrospective study examining the competing risks of progression to end-stage renal disease and death in non-diabetic CKD [8]. Mortality rates from years 1 to 5 for those with stage V CKD were obtained from the Canadian Organ Replacement Register and were estimated to be 15% in year 1 to 57% by year 5 [7].

It was estimated that of all CKD stage III/IV patients, 1.86% receive ESAs for the treatment of anemia based on British Columbia (BC) administrative data; of all individuals with stage V CKD, approximately 77.5% receive ESAs for the treatment of anemia. Those in stage III/IV CKD and those in stage V CKD receiving peritoneal dialysis use subcutaneous ESAs as according to guidelines [9]; and those in stage V CKD receiving hemodialysis are assumed to receive intravenous ESAs. The distribution of patients in stage V CKD on hemodialysis and peritoneal dialysis was also based on administrative data from BC.

### Resource use and costs

Only direct medical costs pertaining to drug acquisition of currently available ESAs in Canada were incorporated in the model. No discounting of future costs was applied. Currently, the only short acting ESA in Canada is Eprex® and the only long acting ESA available is Aranesp®. Based on experiences with biosimilars in the EU, there has been wide variation in the reduced pricing of biosimilars from originator products. In general, price reductions of originator products have also been observed after introduction of biosimilars. In our model, it was assumed that in the base case scenario, epoetin SEBs would be 78% of the originator price (average in EU) when introduced into the Canadian market [10]. Furthermore, it was assumed that upon introduction of an epoetin SEB in the Canadian market, the originator ESA will drop their price by 13% (average in EU) in the base case analysis. During a literature review, we did not find strong evidence suggesting the development or availability of an Aranesp® SEB; however, because the market share and cost of Aranesp® would be impacted by the availability of epoetin SEBs, it was included in the analysis.

The drug cost of anemia treatments were obtained from the Ontario Drug Benefit Formulary [11] and presented as cost per defined daily dose (DDD) according to the World Health Organization [12]. Defined daily dose for epoetin is 1,000 IU while the DDD for darbepoetin is 4.5 mcg. Dosing for anemia treatment is adjusted based on target haemoglobin levels: in the model, median dosages of short acting ESAs were obtained from published sources [13-16] and subcutaneous dosages were calculated to be 30% less than the intravenous dose [9]. We also calculated the dose of long acting ESA based on a conversion ratio of 200:1 from short acting ESA doses less than 8000 IU/week and a ratio of 300:1 for doses greater than 8000 IU/week, due to the curvilinear relationship of epoetin to darbepoetin dosing [17]. We also recognized that there would be drug-related resource utilization costs such as those pertaining to drug storage, drug administration and healthcare personnel wages. These costs were considered to be a part of administrative overhead and were not expected to be significantly different between comparators. Table 1 shows the annual cost of treatment for those with anemia of CKD. It is assumed in the base case scenario that epoetin SEBs will require doses not significantly different from originator drugs in order to achieve the same therapeutic effects, as evidenced by clinical trials of epoetin SEBs [18-20].

**Table 1:** **Costs of treatment for anemia of chronic kidney disease, by disease stage, prior to SEB introduction**

**Treatment strategies for anemia of CKD by disease stage**	**Drug cost per defined daily dose ($)**	**Average dose per week**	**Treatment cost per patient per week ($)**	**Annual treatment cost per patient ($)**
**Stage III/IV short acting ESA (SC)**	14.25	4,186 IU	59.65	**3,101.83**
**Stage III/IV long acting ESA (SC)**	13.36	21 mcg	62.05	**3,226.59**
**Stage V short acting ESA (SC)**	14.25	8,033 IU	114.47	**5,952.45**
**Stage V long acting ESA (SC)**	13.36	27 mcg	79.27	**4,122.01**
**Stage V short acting ESA (IV)**	14.25	11,475 IU	163.52	**8,502.98**
**Stage V long acting ESA (IV)**	13.36	38 mcg	113.71	**5,912.84**

SEB = subsequent entry biologic; CKD = chronic kidney disease; ESA = erythropoiesis stimulating agents; SC = subcutaneous; IV = intravenous; IU = international units; mcg = micrograms.

### Market uptake

Forecasted market uptake rates of epoetin SEBs were based on IMS Health data of overall trends of biosimilar uptake in the EU and projected for five years after introduction onto the market [21]. Biosimilars of ESAs dominate the overall biosimilar market in EU and in general the uptake has been slow due to policies preventing automatic substitution from originator drugs. As ESAs are used for the chronic treatment of anemia, the penetrable market for SEBs consists only of those newly diagnosed with anemia, and existing patients continue to be treated with originator ESAs. The projected uptake rates of epoetin SEB are presented in Table 2. This is assumed to be the uptake rate for epoetin SEBs from market shares of both short acting and long acting originator ESAs; however, the current market share distribution between these two drug categories is not known as some provinces only have one type of ESA and others offer both. The model employs the assumption that the current market share distribution is 50% and 50%, between short and long acting ESAs, respectively. This is a relatively minor assumption, as it does not have any effect on the net budget impact.

**Table 2:** **Projected uptake rates of epoetin SEB in Canada, years 1 to 5**

**Epoetin SEB uptake rates**				
**Year 1**	**Year 2**	**Year 3**	**Year 4**	**Year 5**
1%	4%	7%	11%	14%

SEB = subsequent entry biologic.

### Sensitivity analysis

One-way sensitivity analysis was performed for model input variables using plausible ranges of lower and upper bound estimates, to determine the magnitude of impact each variable has on the model outcomes. A tornado diagram was generated to illustrate the impact of each variable on the budget.

## Results

### Base case

In the base case scenario, the number of expected patients with stage III/IV CKD requiring treatment with ESAs for anemia was 42,644 in the base year (2014). In year 1 the number of patients increased to 49,104; then to 54,919 in year 2; 59,600 in year 3; 62,661 in year 4 and 63,665 in year 5. Given the estimated uptake rate of epoetin SEBs, we expected to have increasing number of SEB users over five years, as shown in Table 3.

**Table 3:** **Expected number of epoetin SEB users in Canada, years 1 to 5**

	**Year 1**	**Year 2**	**Year 3**	**Year 4**	**Year 5**
**Number of SEB users**	982	4,393	8,344	13,785	17,826

SEB = subsequent entry biologic.

Assuming that when epoetin SEBs are introduced into the Canadian market, they will be at 78% of the originator’s price (as the average in EU), that the originator’s price will be reduced by 13% and taking into account the cost of treatment for different stages of CKD, including the number of patients receiving treatment with ESAs or SEBs, the expected annual cost of treatment for years 1 to 5 are as shown in Tables 4, 5, 6, 7 and 8. The ‘Without SEB’ scenario is without the existence of epoetin SEBs and the ‘With SEB’ scenario is with the introduction of epoetin SEBs over the five-year time horizon.

**Table 4:** **Total treatment costs for with and without SEB scenarios in year 1**

**Patient type (CKD stage)**	**Treatment for anemia**	**Year 1 total costs ($)**	
		**Without SEB**	**With SEB**
**Stage III/IV**	**Short acting ESA (SC)**	28,391,013	24,206,178
	**Long acting ESA (SC)**	29,532,964	25,179,805
	**Epoetin SEB**	-	930,614
**Stage V**	**Short acting ESA (SC)**	23,648,751	20,162,925
	**Long acting ESA (SC)**	16,376,488	13,962,594
	**Short acting ESA (IV)**	97,155,486	82,834,767
	**Long acting ESA (IV)**	67,560,443	57,602,033
	**Epoetin SEB**	-	3,193,963
**Total**		**262,665,144**	**228,045,878**

SEB = subsequent entry biologic; CKD = chronic kidney disease; ESA = erythropoiesis stimulating agents; SC = subcutaneous; IV = intravenous.

**Table 5:** **Total treatment costs for with and without SEB scenarios in year 2**

**Patient type (CKD stage)**	**Treatments for anemia**	**Year 2 total costs ($)**	
		**Without SEB**	**With SEB**
**Stage III/IV**	**Short acting ESA (SC)**	31,736,891	25,402,208
	**Long acting ESA (SC)**	33,013,420	26,423,942
	**Epoetin SEB**	-	4,040,419
**Stage V**	**Short acting ESA (SC)**	26,456,982	21,176,169
	**Long acting ESA (SC)**	18,321,156	14,664,254
	**Short acting ESA (IV)**	108,692,464	86,997,449
	**Long acting ESA (IV)**	75,583,082	60,496,699
	**Epoetin SEB**	-	14,292,950
**Total**		**293,803,997**	**253,494,088**

SEB = subsequent entry biologic; CKD = chronic kidney disease; ESA = erythropoiesis stimulating agents; SC = subcutaneous; IV = intravenous.

**Table 6:** **Total treatment costs for with and without SEB scenarios in year 3**

**Patient type (CKD stage)**	**Treatments for anemia**	**Year 3 total costs ($)**	
		**Without SEB**	**With SEB**
**Stage III/IV**	**Short acting ESA (SC)**	35,043,965	26,219,895
	**Long acting ESA (SC)**	36,453,512	27,274,518
	**Epoetin SEB**	-	7,807,525
**Stage V**	**Short acting ESA (SC)**	28,414,572	21,259,783
	**Long acting ESA (SC)**	19,676,765	14,722,155
	**Short acting ESA (IV)**	116,734,773	87,340,957
	**Long acting ESA (IV)**	81,175,581	60,735,569
	**Epoetin SEB**	-	26,863,384
**Total**		**317,499,168**	**272,223,786**

SEB = subsequent entry biologic; CKD = chronic kidney disease; ESA = erythropoiesis stimulating agents; SC = subcutaneous; IV = intravenous.

**Table 7:** **Total treatment costs for with and without SEB scenarios in year 4**

**Patient type (CKD stage)**	**Treatments for anemia**	**Year 4 Total costs ($)**	
		**Without SEB**	**With SEB**
**Stage III/IV**	**Short acting ESA (SC)**	38,308,544	25,996,178
	**Long acting ESA (SC)**	39,849,400	27,041,803
	**Epoetin SEB**	-	13,411,903
**Stage V**	**Short acting ESA (SC)**	29,148,598	19,780,239
	**Long acting ESA (SC)**	20,185,070	13,697,588
	**Short acting ESA (IV)**	119,750,354	81,262,590
	**Long acting ESA (IV)**	83,272,569	56,508,765
	**Epoetin SEB**	-	43,304,392
**Total**		**330,514,535**	**281,003,457**

SEB = subsequent entry biologic; CKD = chronic kidney disease; ESA = erythropoiesis stimulating agents; SC = subcutaneous; IV = intravenous.

**Table 8:** **Total treatment costs for with and without SEB scenarios in year 5**

**Patient type (CKD stage)**	**Treatments for anemia of CKD**	**Year 5 total costs ($)**	
		**Without SEB**	**With SEB**
**Stage III/IV**	**Short acting ESA (SC)**	41,530,100	26,014,455
	**Llong acting ESA (SC)**	43,200,535	27,060,815
	**Epoetin SEB**	-	18,505,171
**Stage V**	**Short acting ESA (SC)**	28,324,361	17,742,380
	**Long acting ESA (SC)**	19,614,295	12,286,394
	**Short acting ESA (IV)**	116,364,164	72,890,512
	**Long acting ESA (IV)**	80,917,865	50,686,950
	**Epoetin SEB**	-	53,556,198
**Total**		**329,951,320**	**278,742,875**

SEB = subsequent entry biologic; CKD = chronic kidney disease; ESA = erythropoiesis stimulating agents; SC = subcutaneous; IV = intravenous.

Table 9 shows the total cost for drugs for the two scenarios for years 1 to 5 and consequently, the incremental budget impact to the Canadian healthcare system from the uptake of epoetin SEBs for treatment of anemia in CKD.

**Table 9:** **Net budget impact due to adoption of epoetin SEBs for years 1 to 5, and cumulative impact over 5 years**

**Year**	**Total drug cost in without SEB scenario ($)**	**Total drug cost in SEB scenario ($)**	**Net budget impact ($)**
**Year 1**	262,665,144	228,045,878	−34,619,266
**Year 2**	293,803,997	253,494,088	−40,309,908
**Year 3**	317,499,168	272,223,786	−45,275,381
**Year 4**	330,514,535	281,003,457	−49,511,077
**Year 5**	329,951,320	278,742,875	−51,208,445
**Cumulative total**	**1,534,434,163**	**1,313,510,085**	**−220,924,078**

SEB = subsequent entry biologic.

### Sensitivity analysis

**Figure 1:**
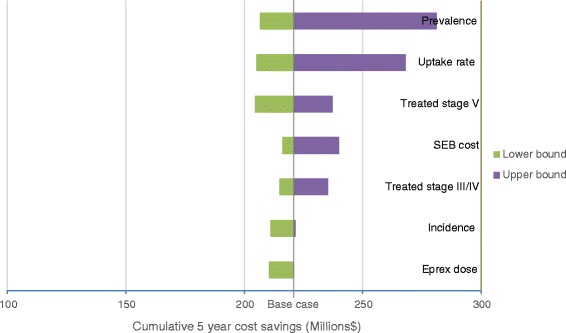
**Tornado diagram demonstrating cost savings based on one-way sensitivity analyses of varying factors.**

One-way sensitivity analyses were performed using +/− 10% margins for incidence of CKD stages III to V; +/− 20% margins for proportion of stage III to IV patients treated with ESAs; and +/− 10% margins for proportion of stage V patients treated with ESAs. Prevalence estimates of CKD stages III to IV vary widely in literature and upper bounds of 8.05% were used [22]. Model results were found to be sensitive to CKD prevalence, as seen in Figure 1. This is simply due to the fact that CKD prevalence has a direct impact on number of patients treated with ESAs. Therefore, the higher the prevalence, the more patients receive ESA treatment, the more cost savings will be brought about if an SEB were to be taken up in the market. The model outcome was sensitive to changes in uptake rate of SEBs. For the uptake rate, we estimated the lower bound to be similar to UK uptake of epoetin SEBs (3% after 5 years), and the upper bound to be similar to Germany’s uptake (43% after 5 years), which resulted in a large range of cost savings. The dose of short acting ESAs was found to trend towards 10% higher with SEBs compared to originators, based on clinical trials in the EU; in clinical studies this was not deemed statistically significant and we also found it to have relatively minor impact on cost savings.

## Discussion

This analysis is the first in Canada to estimate the hypothetical economic impact of ESA use for renal anemia with and without the adoption of epoetin SEBs. It addresses an important and timely question, as epoetin SEBs will likely enter the Canadian market in the near future. Based on our analysis, under market phenomena similar to those seen in the EU, we could expect that Canadian adoption of epoetin SEBs would result in $35 million (year 1) to $50 million (year 5) cost savings annually, with cumulative savings of $221 million after 5 years. This equates to an approximate 14.4% cost difference favoring SEBs. This does not take into account the potential cost savings from use of epoetin SEBs for other indications than management of renal anemia, which was out of the scope of this analysis.

Other published studies have examined the budget impact of epoetin SEBs for the management of chemotherapy-induced anemia in European countries, with positive conclusions [23,24]. Though not directly comparable, Nikolaidi et al. found the budget impact of managing chemotherapy-induced anemia in newly diagnosed cancer patients with epoetin SEBs resulted in cost reductions of at least 10.1%, up to 25.2%, in Greece [23]. Abraham et al. contended that for this patient population, cost savings resulting from the use of epoetin SEBs could substantially increase accessibility to costly neoplastic therapies such as rituximab, bevacizumab, or trastuzumab [24]. Given that the most widely accepted indication of ESAs is in renal disease, and all Canadian provinces publicly fund the use of ESAs for renal anemia, the potential cost savings to the healthcare payer from adopting epoetin SEBs can be reallocated towards expanding access to expensive treatments for patients afflicted with other renal diseases, such as access to rituximab treatment of glomerulonephritis subtypes.

Health Canada recommends against the interchange of originator drugs for its SEB in patients already receiving originator drugs. As such, the estimated uptake rate of chronic medications as ESAs is modest at best. However, as seen in the analysis, even conservative uptake of epoetin SEBs in Canada could result in substantial cost savings to the healthcare payer. Based on our sensitivity analysis, the rate of SEB uptake is the key variable that is amenable to market forces and decision-maker preferences. We believe that due to the mostly positive experience in the EU with epoetin SEBs, there is potential for greater acceptance of its use when introduced into the Canadian market, as such the forecasted uptake could be more dramatic. Furthermore, each jurisdiction in Canada is able to make their own decisions to allow substitution from originator drugs to SEB, thus if the efficacy and safety of epoetin SEBs are sustained and decision-makers feel confident in its adoption, greater cost savings could be realized.

A strength of this budget impact analysis is the incorporation of reliable sources of data to support the cost analysis. We utilized administrative data in BC, which is some of the most comprehensive administrative data in Canada due to availability of a provincial-wide system that captures all direct costs of healthcare utilization, including prescription medications for all residents. However, it appears that the proportion of CKD stage III/IV patients treated for renal anemia with ESAs was low in this jurisdiction, estimated at 1.86% annually. We took the conservative approach and relied on BC data; but, other jurisdictions may have higher estimates. As more clinical data on the efficacy and safety of epoetin SEBs become available, future budget impact analyses should take into account outcomes of treatment with ESAs versus SEBs, such as the cost of avoided blood transfusions. Being that the uptake rate of SEBs appears to be a variable of substantial importance, future research should explore the preferences of stakeholder groups, such as policy makers, hospital administrators, physicians, and patients, for adopting, prescribing, or using SEBs.

## Conclusion

Biologic medicines are among the most costly pharmaceuticals available. It was estimated that biologic drugs accounted for 14% of the pharmaceuticals market in Canada, costing the Canadian healthcare system $3 billion in 2010 [25]. Furthermore, biologics are expected to represent 20% of the pharmaceutical market over the next decade which will result in significant financial pressure on healthcare budgets across the country. The advent of SEBs represents an opportunity to relieve some of the financial pressure, if the efficacy and safety of SEBs can be sustained. Under these assumptions, we have shown that epoetin SEBs for the management of renal anemia can lead to substantial cost savings for the Canadian payer if adopted in the Canadian market. In turn, cost savings can be applied to expand patient access to under-funded treatments for other renal diseases.
